# Five-year lung cancer mortality risk analysis and topography in Xuan Wei: a spatiotemporal correlation analysis

**DOI:** 10.1186/s12889-019-6490-1

**Published:** 2019-02-11

**Authors:** Jinhui Li, Wenbo Guo, Jinjun Ran, Robert Tang, Hualiang Lin, Xiao Chen, Bofu Ning, Jihua Li, Yongchun Zhou, Lung-Chi Chen, Linwei Tian, Yunchao Huang

**Affiliations:** 1 School of Public Health, Li Ka Shing Faculty of Medicine, The University of Hong Kong, Hong Kong, SAR China; 2 Department of Environmental Medicine, New York University, New York, USA; 3 School of Geography and the Environment, University of Oxford, Oxford, OX1 3QY UK; 4 Department of Medical Statistics and Epidemiology, School of Public Health, Sun Yat-sen University, Guangzhou, China; 5 Department of Thoracic Surgery I, Cancer Research Institute of Yunnan Province, The Third Affiliated Hospital of Kunming Medical University (Yunnan Cancer Hospital), Kunming, Yunnan China; 6 Xuanwei Center for Disease Control and Prevention, Xuanwei, Yunnan China; 7 Qujing Center for Disease Control and Prevention, Qujing, Yunnan China; 8 Cancer Research Institute of Yunnan Province, The Third Affiliated Hospital of Kunming Medical University (Yunnan Cancer Hospital), Kunming, Yunnan China

**Keywords:** Xuan Wei, Lung cancer, Mortality risk, Coal mine, Spatiotemporal correlation

## Abstract

**Background:**

In Xuan Wei, China, the lung cancer mortality rate is rising significantly more than that of the nation overall. However, it remains unclear 1) if improved diagnosis can just partially explain this observation and how other local risk factors may be correlated with the lung cancer mortality rate and 2) how the lung cancer mortality rates differ within Xuan Wei and how these spatiotemporal patterns are linked with local risk factors. To increase etiological knowledge, this study evaluated the spatial and temporal distributions of the health effects (the lung cancer mortality rates) from 2011 to 2015.

**Methods:**

Four steps of spatial analysis were applied, as follows: 1) hotspot analysis to determine the geographical patterns of lung cancer mortality, 2) spatially-weighted sum to identify areas with higher health risks, 3) bivariate statistical analysis to assess the overall correlation between coal mines and lung cancer mortality, and 4) geographically-weighted regression to test for correlations among different towns within Xuan Wei.

**Results:**

Women had higher lung cancer mortality rates than those in men, with an increasing trend in both sexes over time. The incidence rates in Laibin Town were the highest in Xuan Wei every year. Over the 5-year study period, the lung cancer mortality was increasingly concentrated in Laibin, Shuanglong, and Longchang, where the smoky coal mines are most concentrated. The population-level health risks from the coal mine in Xuan Wei were mapped and divided into five types of risk areas (Type I – Type IV). Correlation analysis revealed that there was no significant correlation between lung cancer mortality as a whole and coal mine distribution during the 5-year study period. However, the geographically-weighted regression revealed a stronger correlation in medium (Type III) and second-lowest (Type IV) health risks.

**Conclusions:**

Xuan Wei lung cancer mortality has increased continuously since the third national retrospective surveys on the causes of death by the Ministry of Health of the People’s Republic of China (2004–2005), especially for local women and residents over 35 years of age. Geographically, lung cancer in Xuan Wei showed unique spatiotemporal clustering. The local lung cancer mortality was significantly correlated with the smoky coal mine geographically. Some specific towns (Laibin, Shuanglong, and Longchang) within Xuan Wei manifested high correlations between lung cancer mortality and coal mines. The effects of coal mines on lung cancer mortality rates also spread geographically outward from these areas. Public health concern regarding lung cancer in Xuan Wei should prioritize higher-risk towns surrounded by smoking coal mines. Intervention strategies for particular toxic coal types require further studies on their chemical characteristics and mechanisms of carcinogenesis. Additional studies are also warranted to systematically examine the local environmental health risks related to coal industries and combustion air pollution and eventually to conduct early screening of lung cancer for local people who are more exposed to smoky coal in high-risk areas.

## Background

According to the World Cancer Report of the World Health Organization (WHO), lung cancer is the leading cause of mortality in the world [1]. In 2012, approximate 1.6 million people died due to lung cancer. For men, lung cancer is the most common cancer worldwide [2], with an age-standardized mortality rate (ASMR) of 33.8 per 100,000. It is the fourth most lethal cancer for women, with an ASMR of 13.5 per 100,000 [3]. Most people with lung cancer are diagnosed at late stages, which directly affects the survival rate [4]. From 1999 to 2006, the 5-year survival rates of lung cancer for men and women were 14 and 19% respectively. Taking into account the geographic locations of lung cancer deaths in different regions, the countries in the WHO Western Pacific Region (WPRO) have the largest number of deaths (48% in men, 45% in women), and it is predicted that the number of lung cancer mortality cases will reach three million in 2035 [2].

In China, lung cancer accounts for the largest number of cancer cases in both sexes and is the top cancer mortality risk for men. In 2010, the morbidity and mortality of lung cancer in China reached 46.08/100,000 and 37.00/100,000, respectively [5]. Statistically, lung cancer occupied almost 35.7% of newly diagnosed cases and 37.56% of cancer deaths in 2012. Lung cancer mortality rates are generally higher among women in urban areas, peaking at 80+ years of age [6]. Moreover, more than half of the world population and most rural populations in China still use solid fuels such as coal for cooking and heating. This is listed as one of the top five risk factors to public health with 3·5 million (2.7 million to 4.4 million) deaths and 4.5% (3.4–5.3%) of global disability-adjusted life-years (DALYs) [7].

**Fig. 1:**
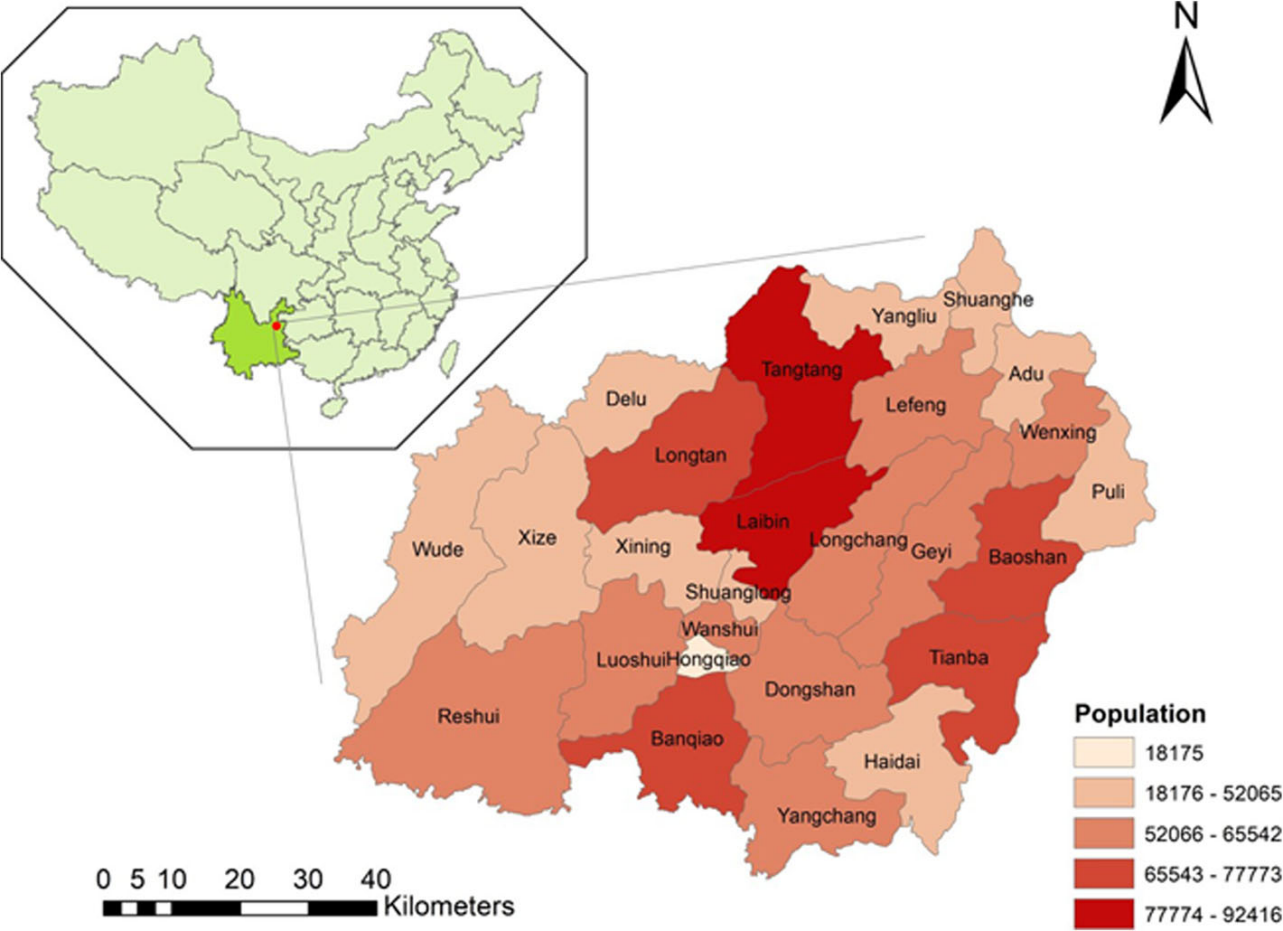
Location and population distribution of Xuan Wei

Xuan Wei, a county-level city in Yunnan province, China, has experienced high mortality rates since 1973–1975. According to the first national mortality survey, the lung cancer mortality rates in Xuan Wei are 27.7/100,000 for men and 25.3/100,000 for women, almost five times that of China’s national average (4.97/100,000 for both sexes) [8, 9] (Fig. 1). Since 1980s, potential suspected factor of tobacco smoking has been denied by researchers. Because in Xuan Wei most man are smokers and few woman smoke there [10, 11]. Previous studies concluded that indoor air pollution, derived from household coal combustion, could be the cause of local lung cancer occurrence [12], which contributed to the International Agency for Research on Cancer (IARC) declaring it a Group I carcinogen to humans. Although interventions such as stove improvements have been carried out, the local lung cancer deaths have continued to increase in the past three national mortality surveys [10, 13]. Previous studies have explored the relationship between lung cancer mortality rates and carcinogenetic chemicals in different coals that patients had burned or in discharged coal combustion soot that might not diffuse far away due to topology and wind direction [14–16]. However, studies on the geological distribution of geochemical perturbation and mine locations at spatial scales in Xuan Wei are limited [17, 18]. Therefore, knowing more about the spatial, temporal, and spatiotemporal distributions of lung cancer in Xuan Wei could provide clues to inform public health priority and prevention to improve the health of Xuan Wei residents.

Spatial-temporal analysis can add references to risk factor exposure and help to assess the effectiveness of existing health interventions among Xuan Wei residents, making these analyses valuable tools to improve public health quality and better distribute financial and medical resources [19]. Based on the most recent cancer registration data in Xuan Wei (2011–2015), we conducted a Geographic Information System (GIS)-based spatiotemporal analysis, investigated the spatial and temporal clustering of lung cancer in Xuan Wei, and identified the health effects associated with the spatial distributions due to coal mines in Xuan Wei. Our findings will help to better understand the locational characteristics of cancer mortality in Xuan Wei and the impact of environmental factors, as well as help to prioritize the provision of suitable healthcare to the local residents.

## Methods

### Sampling site

**Fig. 2:**
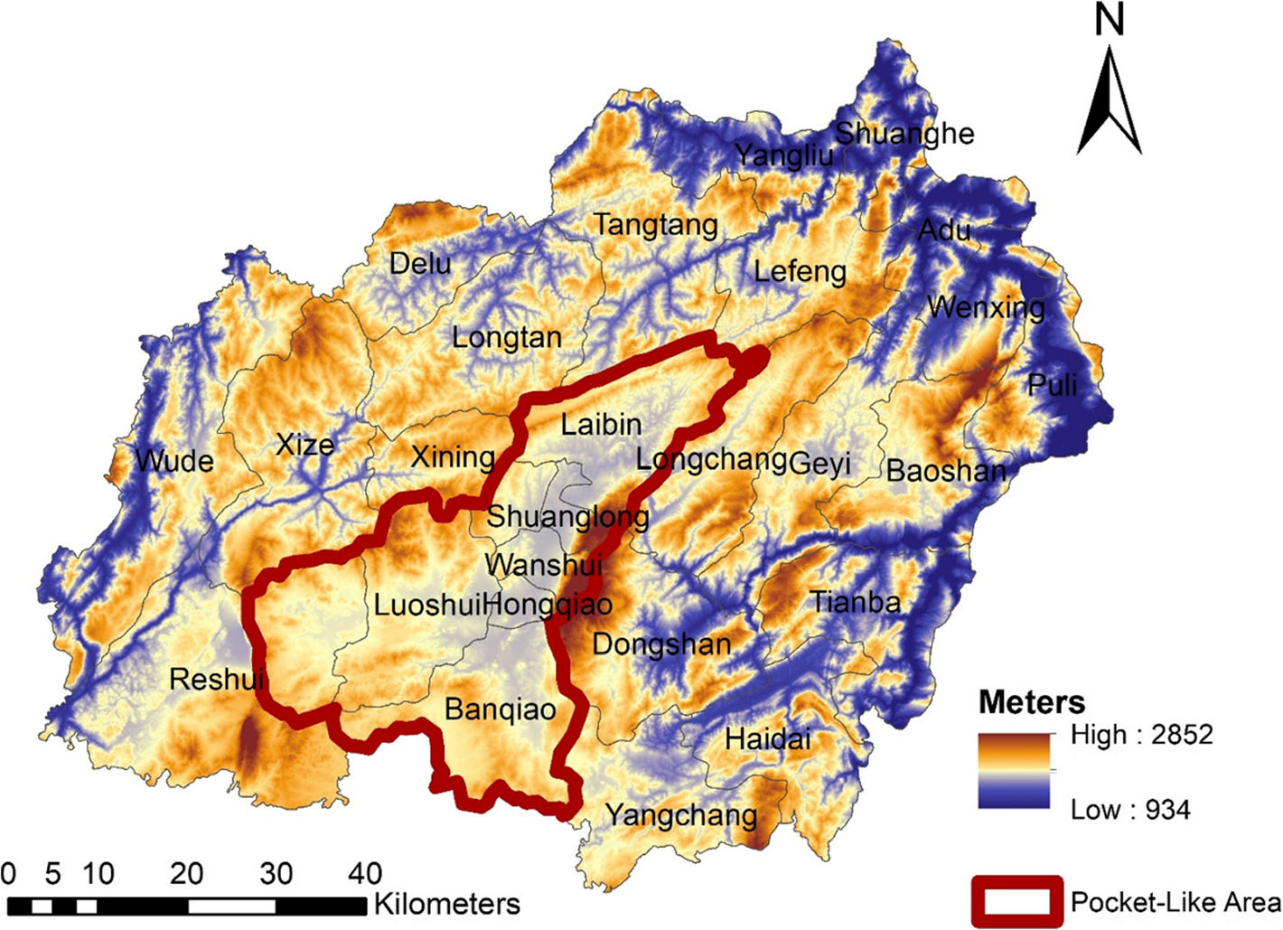
Physical features of Xuan Wei [18]

The study area is located in northeast Yunnan Province, China (103 °30′- 104° 40′E, 25 °3′-26°41’N) (Figs. 1, 2) [18]. Xuan Wei belongs to the slope region of the transition zone of the Yunnan-Guizhou Plateau. In 2015, the population reached 1,529,463 and encompassed 606,988 m^2^ within 28 towns. The majority of the local people are traditional farmers and the population structure is stable. This study focused on information based on the town level, including a total of 344 villages. The name for each town is the town-governed region that includes all its incorporated villages.

### Data collection

Lung cancer mortality data from 2011 to 2015 were obtained from the online reports of the death registration system (DRS) in the Xuan Wei Center for Disease Control and Prevention (XWCDC). The National Central Cancer Registry (NCCR) of China, established in 2012, is a national systematic management and information source for cancer surveillance. Each CDC is responsible for producing standardized death certification cards and reporting information to the higher administrative regions through the DRS [5]. All deaths should be coded based on the 10th version International Classification of Disease (ICD) guidelines, which codes lung cancer as C33-C34. The death certification card primarily includes personal, clinical, and cause of death information. To avoid report bias and missing bias, village doctors verify these cards by interviewing the family members of the deceased and higher corresponding health division also check them as well. The details have been previously described by our team [13]. The age- and sex-specific populations for each town in Xuan Wei were extracted from the Annual Statistical Report of Xuan Wei for each year. Capture and recapture methods were conducted to confirm some under-reported rates of death as a standard approach to estimate the completeness of registries and true incidence rates of diseases [20, 21].

### Statistical analysis

We used R 3.1.3 (R Development Core Team, 2015) and MATLAB (R2016a version, MathWorks Inc., Natick, USA) to perform descriptive analyses. The crude mortality rates and their distributions were first explored. The sex- and age-specific mortality rates were adjusted by the China National 6th census population (2010) and the WHO World standard population, respectively. We used percentages to show the cumulative risk of lung cancer before 60 years of age among Xuan Wei residents. The truncated age-standardized rate was used to further explore the age-specific rates in the elderly [22]. We also compared the age- and sex- specific mortality rates in one community with an especially high mortality rate compared to the overall mortality rate of Xuan Wei. Statistical significance was examined using a Poisson model, and two-tailed *p-*values less than 0.05 were considered statistically significant.

### Spatial analysis

**Fig. 3:**
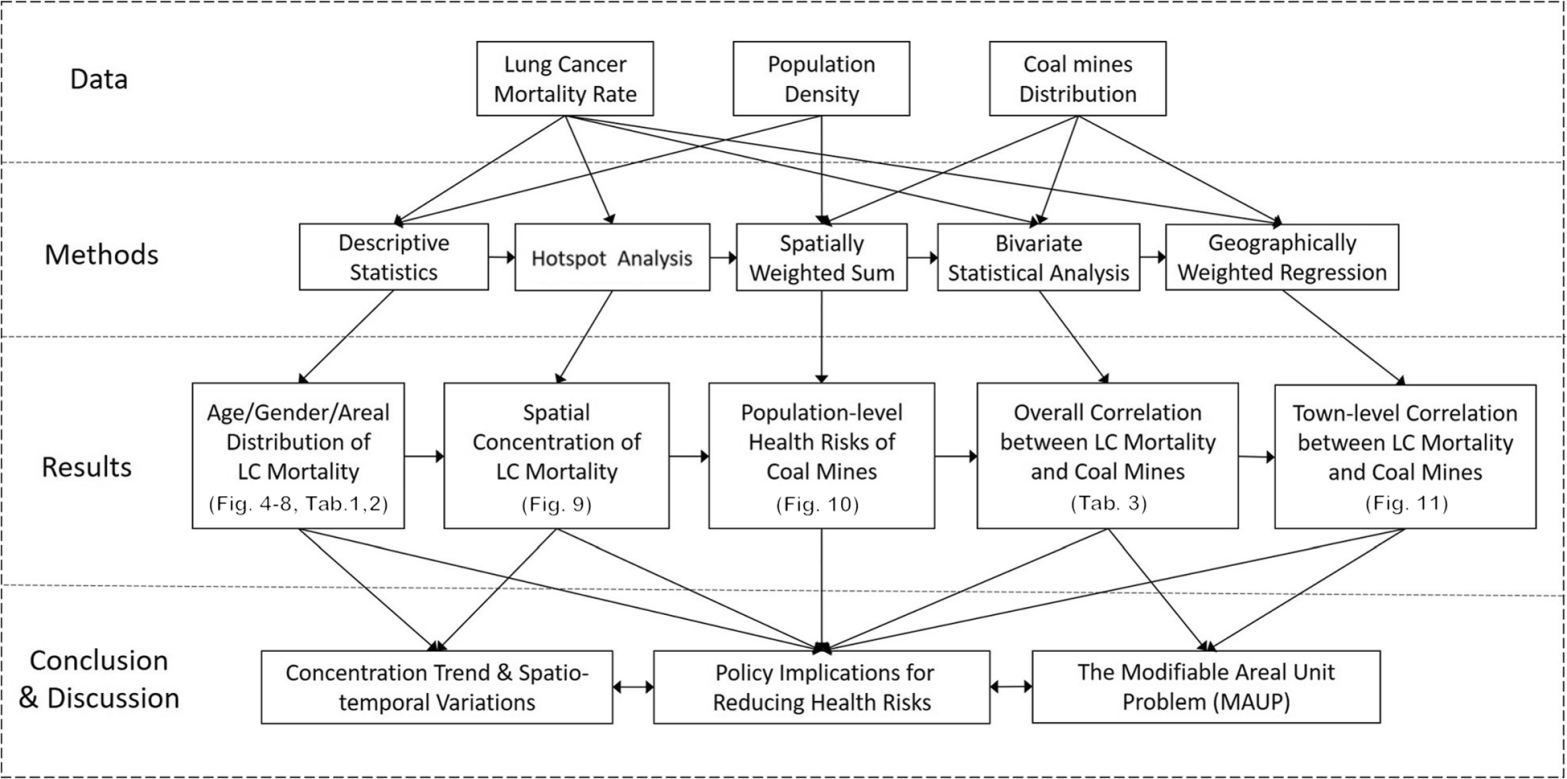
Operational research framework

ArcGIS 10.5 (ArcGIS 10.5 version, Esri Inc., California, USA) was utilized for the spatial analyses. Four categories of analysis tools were used to assess the spatiotemporal patterns and correlations, including hotspot analysis, spatially-weighted sum, bivariate statistical analysis, and geographically-weighted regression (GWR). As shown in the operational research framework (Fig. 3), hotspot analysis was first used to identify the geographical patterns of lung cancer mortality, after which the spatially-weighted sum was applied to cluster and map the population-level health risks of different towns within Xuan Wei by overlapping the population density and coal mine distributions. The next two steps were designed to examine the spatiotemporal correlations between coal mine distributions and lung cancer mortality: the bivariate statistical analysis was used to assess the overall correlation, while the GWR was used to more deeply investigate these potential correlations for different towns.

Hotspot analysis was conducted to map the geographical concentration patterns of lung cancer mortality. The temporal variations and group differentiations were also examined through the 5-year study period [23]. This tool calculates the Getis-Ord Gi* statistic for each feature in a dataset and to find statistically significant hotspots, which have high values and are surrounded by other features with high values [24]. The resulting *Z*-score indicates where features with either high or low values cluster spatially. The Getis-Ord local statistic is given as

1 $$ {\mathrm{G}}_{\mathrm{i}}^{\ast }=\frac{\sum_{j=1}^n{w}_{i,j}{x}_j-\overline{X}{\sum}_{j=1}^n{w}_{i,j}}{\sqrt[S]{\frac{\left[n{\sum}_{j=1}^n{w}_{i,j}^2-{\left({\sum}_{j=1}^n{w}_{i,j}\right)}^2\right]}{n-1}}} $$

Where *x*_*j*_ is the attribute value for feature j, w_*i*, *j*_is the spatial weight between feature i and j, n is equal to the number of features, and:

2 $$ \overline{X}=\frac{\sum_{j=1}^n{w}_j}{n} $$

3 $$ S=\sqrt{\frac{\sum_{j=1}^n{w}_j^2}{n}-{\left(\overline{X}\right)}^2} $$

Secondly, the spatially-weighted sum was adopted to evaluate the population-level health risks from coal mines [25], which is a classical way presenting the interactive outcomes of two or more factors by adding the weights of the elements from one layer to another layer with area-attributes [25]. The weights of the coal mine distribution within the area were added to the population layer based on the population density.

Thirdly, the bivariate statistical analysis was used to examine the spatial interrelation between coal mines and lung cancer mortality [26]. This method allows the exploration of relationships between two or more different types of attribute data. Before we began the analysis, the polygon data were transferred into raster data at resolution of 30 min. Finally, a GWR model was employed to further explore how geography was contributing to the associations of the coal mine locations with lung cancer mortality for different areas of Xuan Wei [24]. The spatially heterogeneous processes were modelled to examine the links between dependent and independent variables, treating the spatial attributes as non-negligible contributors [22]. The equation for a typical GWR of the ordinary least-square regression (OLS) model is as follows:

4 $$ {\mathrm{y}}_i\left(\boldsymbol{u}\right)={\beta}_{0i}\left(\boldsymbol{u}\right)+{\beta}_{1i}\left(\boldsymbol{u}\right){x}_{1i}+{\beta}_{2i}\left(\boldsymbol{u}\right){x}_{2i}+\dots +{\beta}_{mi}\left(\boldsymbol{u}\right){x}_{mi} $$

Where the dependent variable *y* was defined as the lung cancer mortality rate and the independent variable *x* as the productivity of the coal mine. The notation *β*_0*i*_(***u***) indicates that the parameter describes a relationship around location *u* and is specific to that location. For each of the n observations in the dataset, a measurement of its position was available in a spatial coordinate system.

## Results

### Demographic and mortality analysis

There were a total of 5789 lung cancer deaths in Xuan Wei between 2011 and 2015, including 2387 women and 3402 men. As shown in Table 1, the crude mortality rates were 70.78, 80.27, 78.45, 80.30, and 75.71 per 100,000 each year. The age-standardized rates were calculated by 6th Chinese population census data (CASMR) and world standard population data (WASMR) separately. The cumulative rates of mortality from 0 to 60 years of age ranged from 17.01 to 22.30%. The average annual truncated age-standardized rate (TASR) of lung cancer in Xuan Wei from 2011 to 2015 was 81.96 per 100,000.

**Table 1:** Lung cancer incidence in Xuan Wei, China

Year	No. of Cases	Crude rate (1*10^5^)	CASIR (1*10^5^)	WASIR (1*10^5^)	Cum Rate 0~60(%)	TASR(35–60) (1*10^5^)
2011	1057	70.78	40.34	34.56	21.43%	86.33
2012	1211	80.27	44.32	37.96	22.30%	90.13
2013	1160	78.45	40.82	34.90	19.27%	78.72
2014	1228	80.30	41.85	35.73	20.03%	82.22
2015	1158	75.71	39.34	33.78	17.01%	72.39

* CASIR, age-standardized rate (China 6th population census 2010); WASIR, age-standardized rate (World standard population): Cum Rate, accumulative rate; TASR, truncated age-standardized rate (World standard population)

**Fig. 4:**
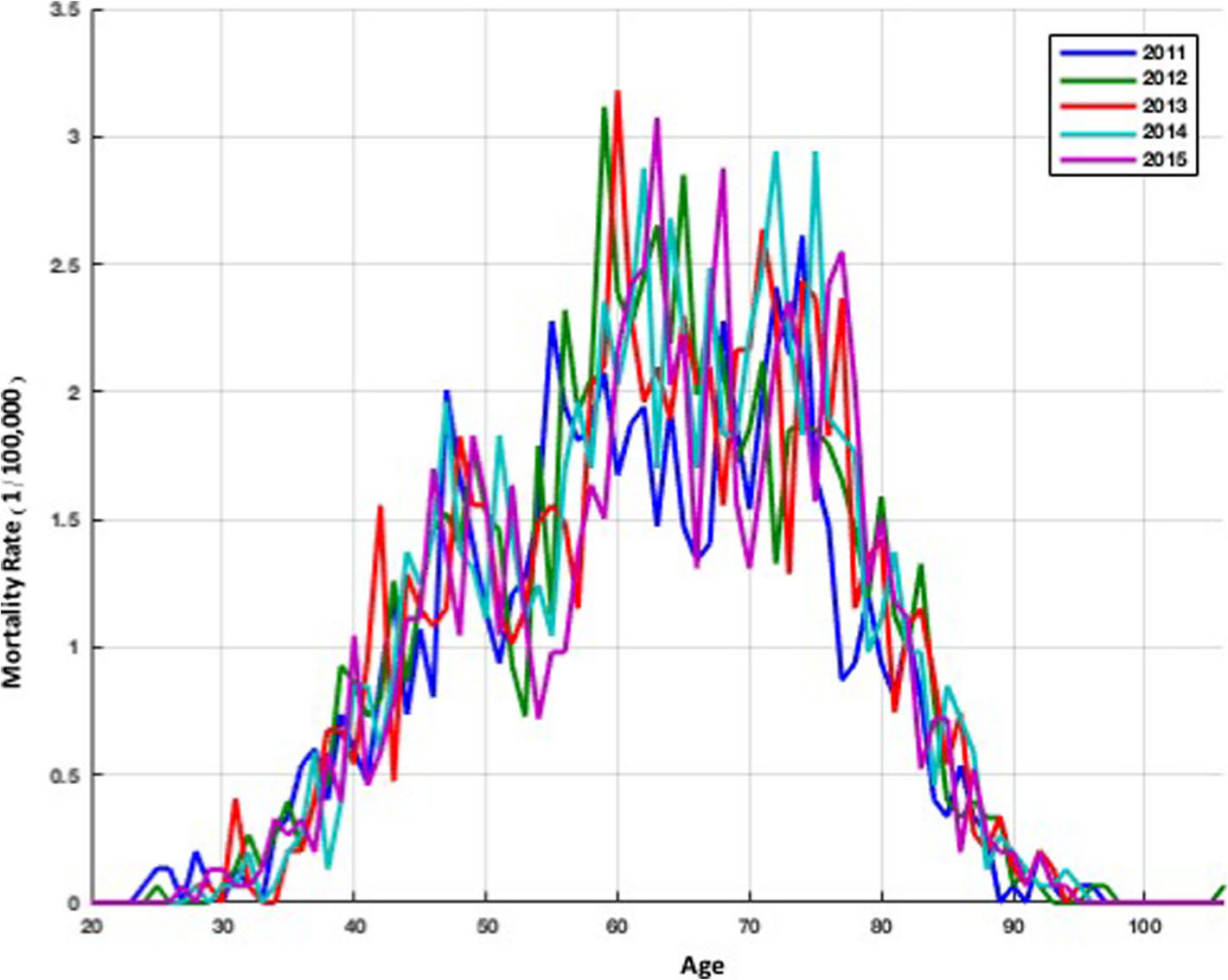
Distribution of lung cancer incidence in Xuan Wei, 2011–2015

**Fig. 5:**
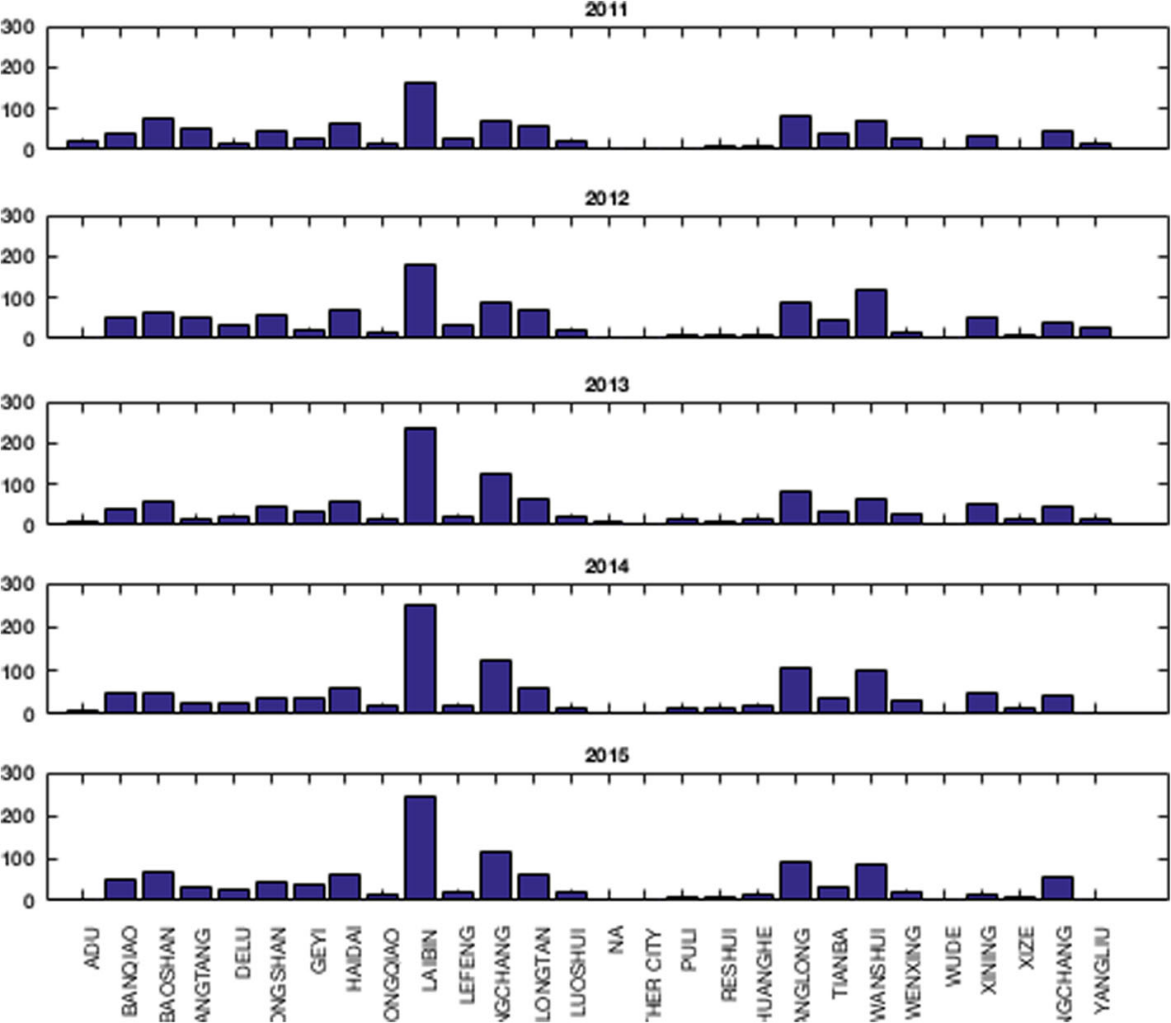
Distributions of town-level lung cancer mortality cases in Xuan Wei

**Fig. 6:**
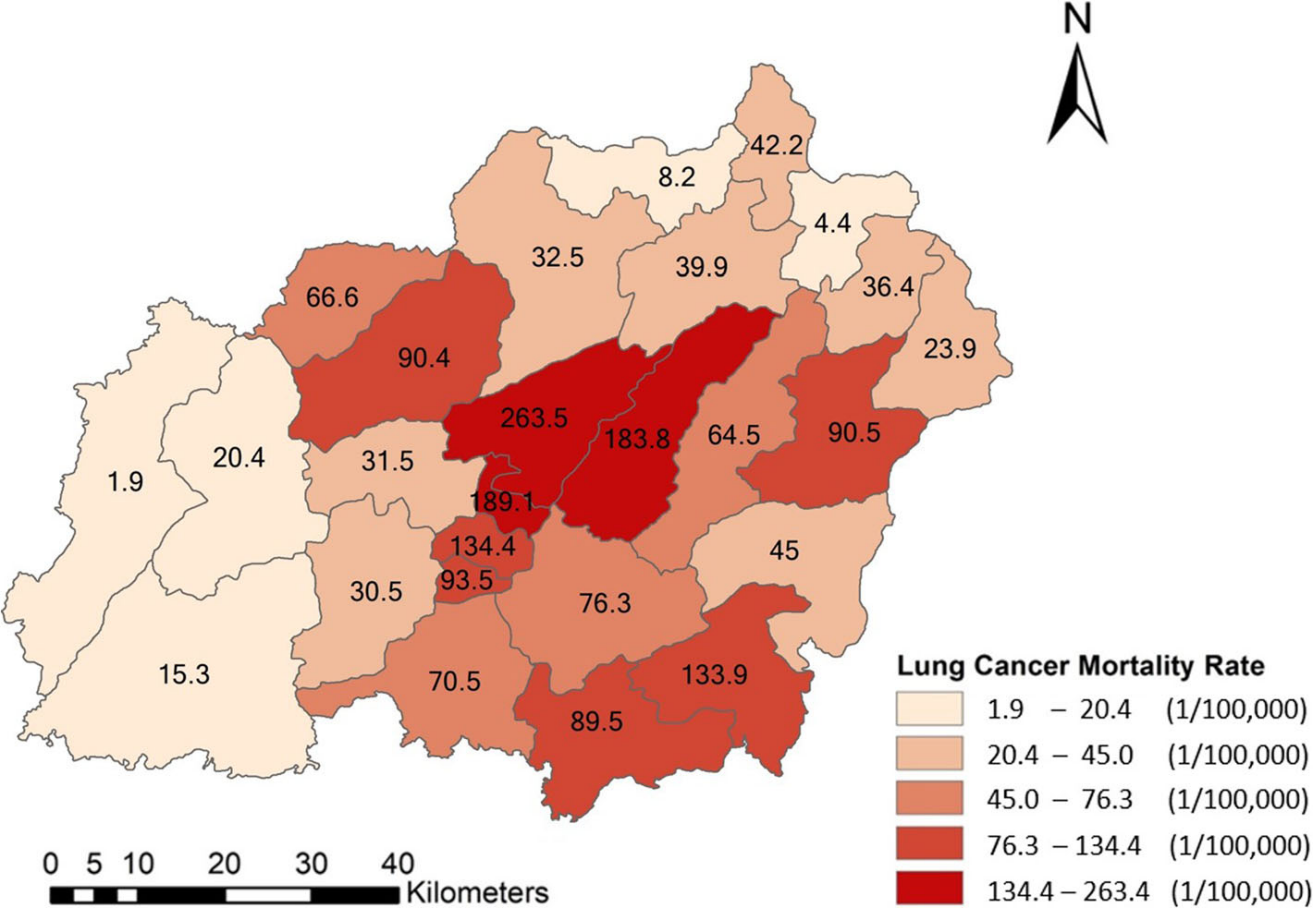
Distributions of lung cancer mortality rates in Xuan Wei, 2011–2015

**Fig. 7:**
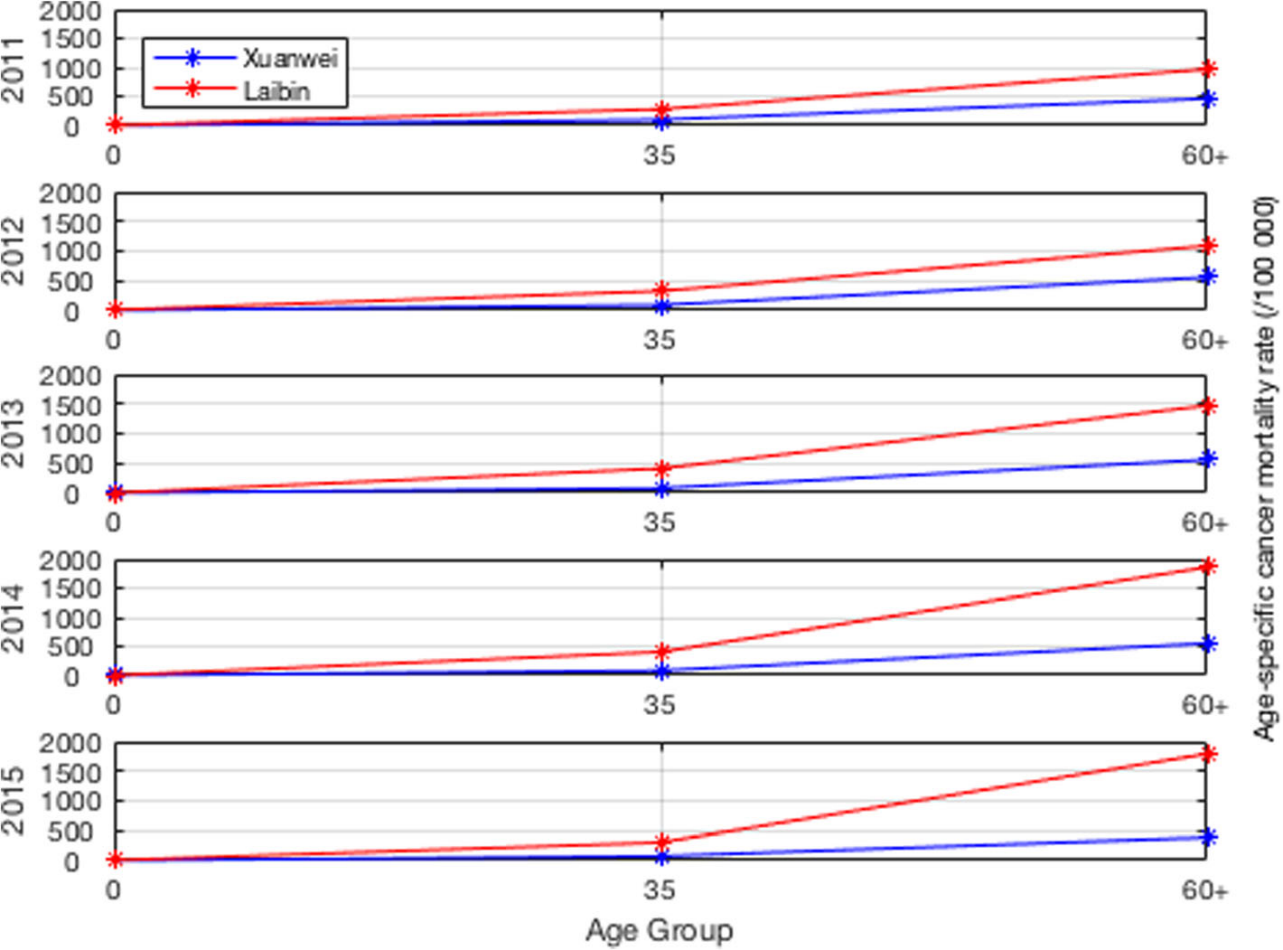
Age-specific mortality rates for lung cancer in Xuan Wei and Laibin Town

**Fig. 8:**
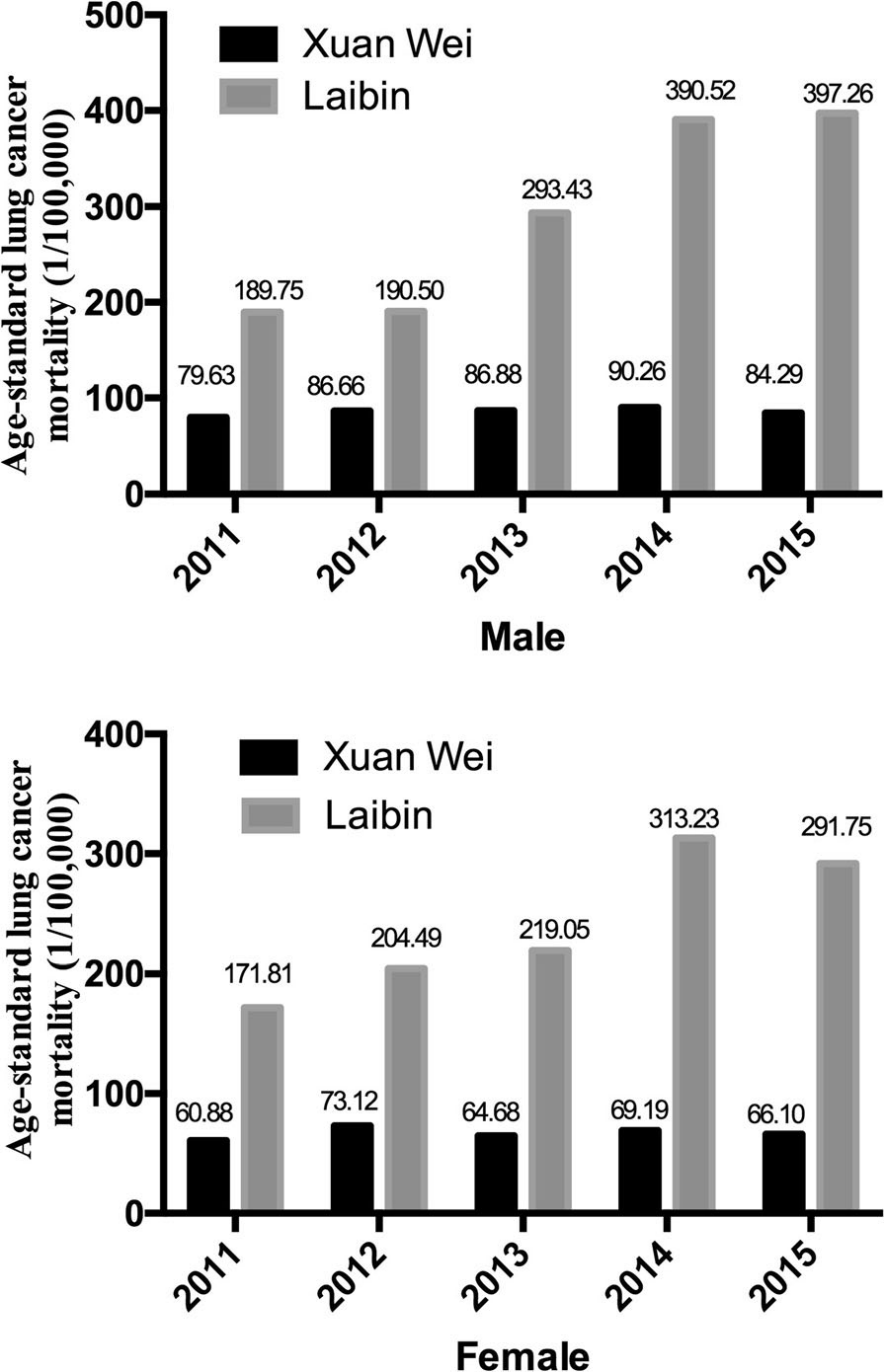
Age-standard lung cancer mortality for both sexes in Xuan Wei and Laibin Town

The total number of deaths was higher in men than that in women. The peak age for lung cancer mortality in Xuan Wei was between 58 and 64 years (Fig. 4). The town-level lung cancer mortality case distributions from 2011 to 2015 and the average lung cancer mortality rates are shown in Figs. 5 and 6, respectively. Among the 28 towns in Xuan Wei, Laibin, Shuanglong, and Wanshui had the highest lung cancer mortality rates (34.10% of the total mortality cases), seven towns showed relatively higher mortality rates (34.50% of the total mortality cases), eight towns were medium-endemic (20.85% of the total mortality cases), five towns had lower mortality rates (7.13% of the total mortality cases), and the remaining three towns had the lowest lung cancer mortality rates in Xuan Wei (1.80% of the total mortality cases). Laibin town had the largest number of lung cancer mortality cases each year, reaching as high as 9.98% of whole cases. Therefore, we compared the age-specific mortality rates between Laibin and Xuan Wei overall for both men and women during these 5 years. As shown in Fig. 7, when the local population was over 35 years of age, the lung cancer mortality was much higher in Laibin town compared to the average lung cancer mortality rate for all of Xuan Wei. Especially in the past 3 years, there was an increasing trend in lung cancer mortality in the Laibin population among those over 35 years of age. Moreover, the sex-specific lung cancer age-standardized rate mortalities also differed significantly between Laibin and Xuan Wei. As Fig. 8 indicates, the mortality rates had similar patterns in both men and women. However, the rates in Laibin were approximately twice those of Xuan Wei in 2011 and 2012. The mortality rates increased to approximately four times for men and three times for women from 2013 to 2015 compared to the average for Xuan Wei during the same time period.

We further explored the variations and changes in lung cancer mortality rates during the study period by analysis of variance (ANOVA). Table 2 shows the significance of the changes during the 5-year period. There was a significant change (*P* = 0.039) from 2011 to 2012 and a relatively notable significance (*P* = 0.166) from 2014 to 2015. However, the changes between 2012, 2013, and 2014 were not statistically significant.

**Table 2:** Analysis of variance results

	LC mortality 2011	LC mortality 2012	LC mortality 2013	LC mortality 2014	LC mortality 2015
LC mortality 2011	–	0.039	0.226	0.172	0.395
LC mortality 2012	0.039	–	0.751	0.166	0.444
LC mortality 2013	0.226	0.751	–	0.504	0.549
LC mortality 2014	0.172	0.504	0.261	–	0.166
LC mortality 2015	0.395	0.444	0.549	0.166	–

### Spatial-correlation analysis

**Fig. 9:**
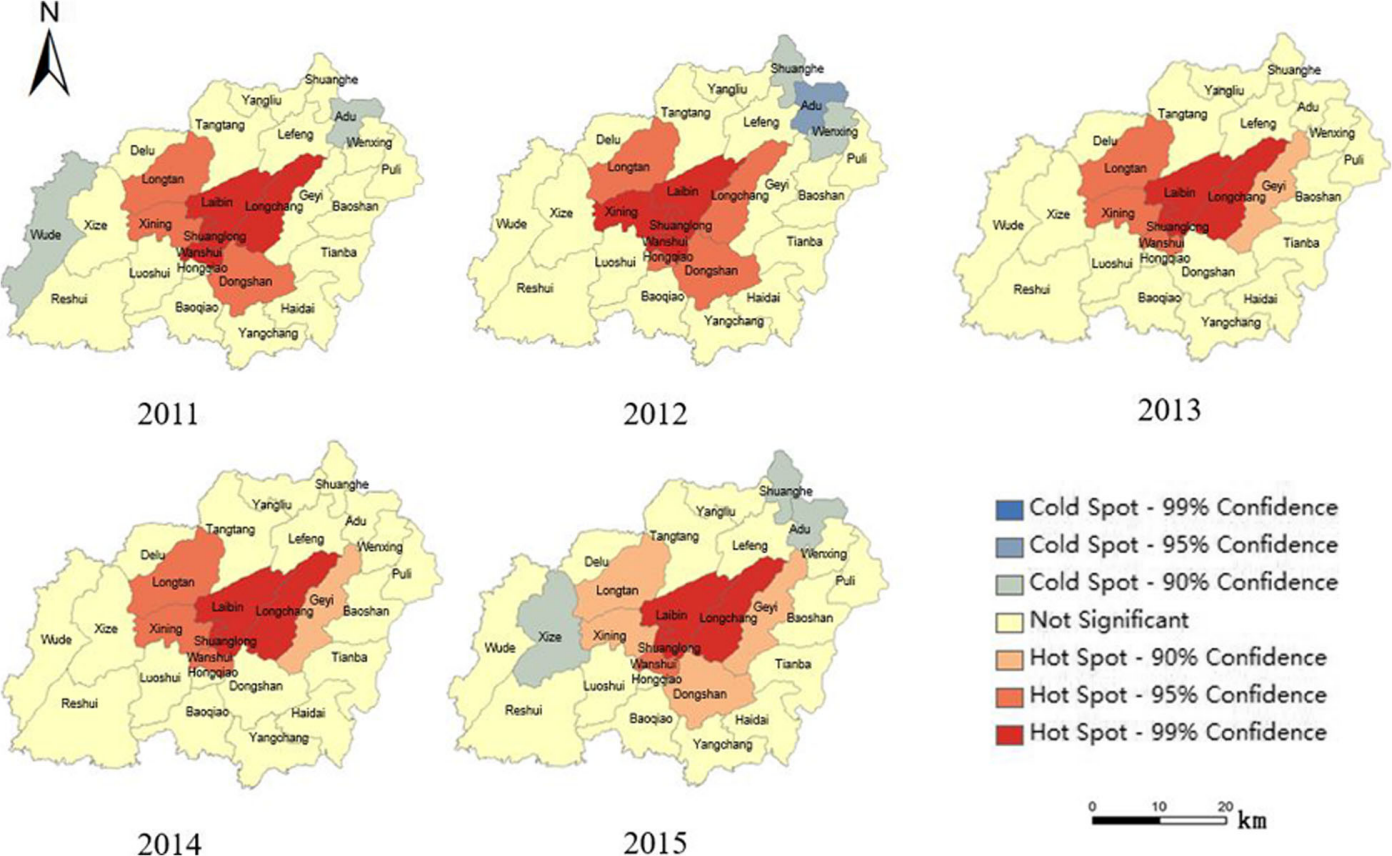
Hotspot analysis of lung cancer mortality in Xuan Wei

The hotspot analysis based on Getis-Ord Gi* revealed a concentration trend of lung cancer mortality during the study period (Fig. 9). The hotspots were mainly concentrated at the ravine of the cuneiform area around towns of Laibin, Shuanglong, and Longchang, where heavy coal mines were located and which were also areas with high population densities. Laibin, Shuanglong, and Longchang were surrounded by secondary and less-significant clusters of hotspots. At the same time, there were also spatiotemporal variations in hotspot development. The hotspot analysis illustrated a decreasing confidence interval for most towns around Laibin, which indicated that the lung cancer mortality was more concentrated over the 5-year study period. Several cold spots (geographical concentrations of lower lung cancer mortality rates) were detected at Adu and Wude towns in 2011 and 2012. No significant cold spots were observed across Xuan Wei in 2013 and 2014 but were observed again in 2015. All cold spots demonstrated a weak confidence of 90%.

**Fig. 10:**
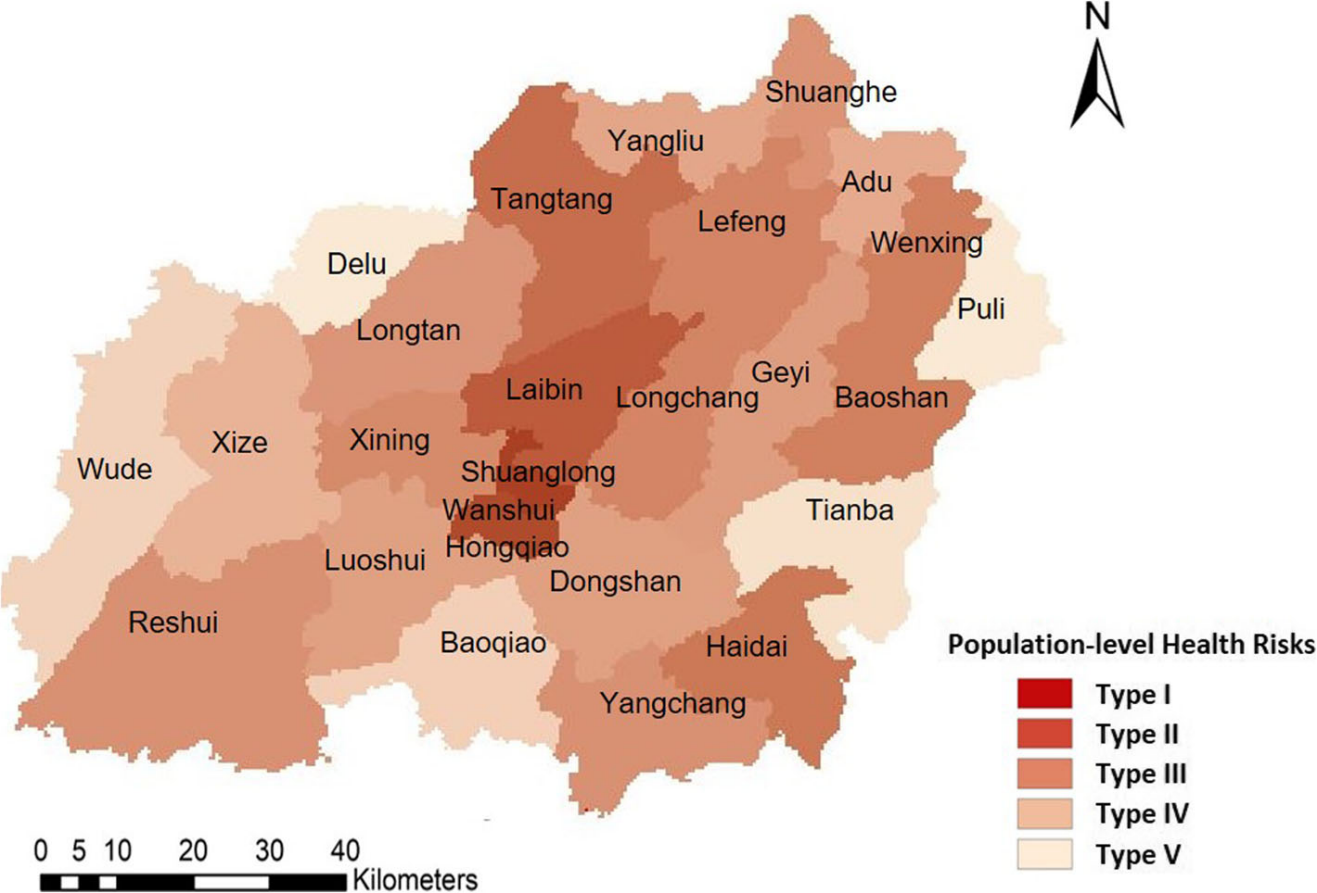
Population-level health risks assessment based on spatially-weighted sum

Spatially-weighted sum from overlapping the population densities and distributions of local coal mine can be used to identify the health risks from coal mines (Fig. 10). The rasters of the population densities and coal mines were used in the overlap analysis. The weights of the coal mine raster were calculated using the annual productivity, which was considered an important indicator of the density distribution of the coal mine. The health risk of each area was calculated based on the summation of the production value of the coal mines within the area. The result of the spatially-weighted sum indicates the population-level health risks in five categories. The five categories were divided using the Jenks natural breaks default in ArcGIS. Natural break classes are based on natural groupings inherent in the data. Class breaks are identified that best group similar values and that maximize the differences between classes. Shuanglong and Wanshui towns had the highest health risks (Type I) from the coal mine, as both their populations and coal mines were geographically concentrated. Laibin was the only area with the second highest-level health risks from coal mines (Type II). The medium-level health risks were distributed close to the geometric centre of Xuan Wei and also crossed areas further from the geometric centre, including the towns of Longchang, Tangtang, Haidai, Wenxing, Baoshan, Reshui, etc. The second lowest health risks (Type IV) were distributed in areas further from the geometric centre, including the towns of Geyi, Lefeng, Dongshan, Xize, Longtan, etc. The outskirts of Xuan Wei experienced the lowest levels of health risks (Type V) from the coal mine. The spatiotemporal variations observed in 2015 indicated increased health risks in Luoshui town. This was due to the inner-city (migration from other areas of the city) and inter-city migration (migration from other cities): Luoshui has seen a significant increase in population growth, from 44,212 in 2011 to 59,006 in 2015.

Based on the analysis of the geographical patterns of lung cancer mortality and health risks from the coal mines, we examined the spatial correlation between health risks and lung cancer mortality. First, bivariate statistical analysis was employed to examine the overall spatial correlation of the raster layers of coal mine distributions and lung cancer mortality from 2011 to 2015. A correlation matrix was then calculated (Table 3). The results showed a statistically significant temporal correlation and continuity, as well as temporal variation in lung cancer mortality from 2011 to 2015. The lung cancer mortality demonstrated a high correlation across the 5-year span. The spatial correlation between lung cancer mortality and coal mine distribution was not significant compared to the interrelations of lung cancer mortality during the 5 years; however, slight changes were observed. The correlation decreased from 0.242 in 2011 to 0.180 in 2013 and climbed to 0.203 in 2015. A follow-up analysis was then conducted to identify the spatiotemporal correlation at a smaller spatial scale.

**Table 3:** Correlation matrix of the spatial correlation analysis

Layer	LC mortality 2011	LC mortality 2012	LC mortality 2013	LC mortality 2014	LC mortality 2015	Coal mine
LC mortality 2011	1.000	0.944	0.888	0.836	0.924	0.242
LC mortality 2012	0.944	1.000	0.905	0.832	0.901	0.204
LC mortality 2013	0.888	0.905	1.000	0.931	0.946	0.180
LC mortality 2014	0.836	0.832	0.931	1.000	0.922	0.193
LC mortality 2015	0.924	0.901	0.946	0.922	1.000	0.203
Coal mine	0.242	0.204	0.180	0.193	0.203	1.000

**Fig. 11:**
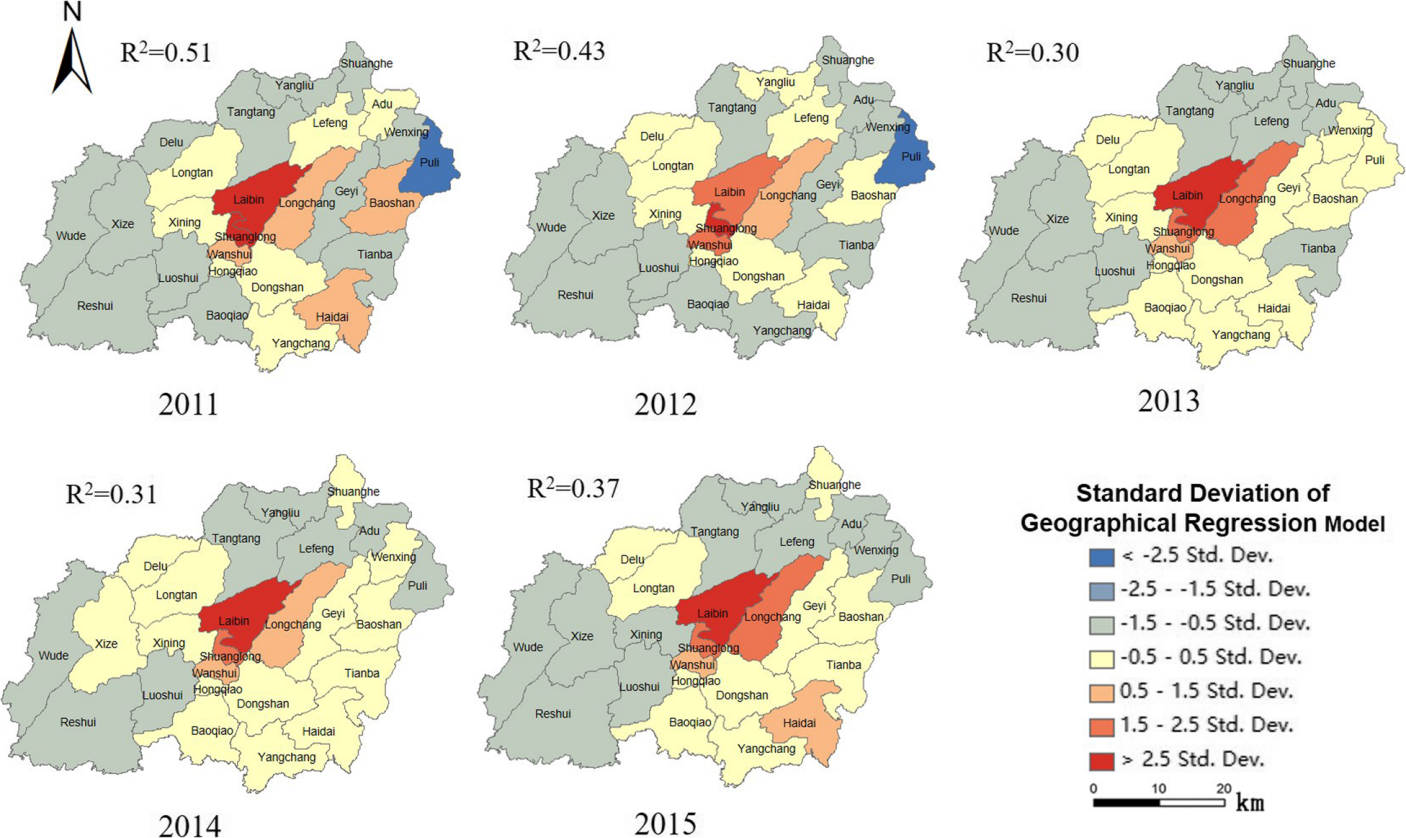
Geographically-weighted regression

A GWR model was employed to examine the association between the locations of coal mines and increased lung cancer mortality in Xuan Wei, as shown according to the standard deviations in different towns (Fig. 11). We ran the GWR (bandwidth set to 0.79) after the linear regression was passed through the T-test. The r-squared value of the GWR indicated a decrease from 0.51 in 2011 to 0.30 in 2013 and an increase to 0.37 in 2015. This was due to an increasing concentration of lung cancer mortality around Laibin from 2012 to 2014. The results showed that the association between coal mines and lung cancer mortality rates were most significant for medium (Type III) and second-lowest (Type IV) health risk areas, which is geographically closer to where smoky coal mine were most concentrated. In addition, although the lung cancer mortality showed a concentrated pattern around the towns of Laibin, Shuanglong, and Longchang, the associations of coal mines with lung cancer mortality in these areas were comparatively lower than those in other areas in Xuan Wei. The changes in r-squared value in coefficient analysis indicated that the effects of coal mines on lung cancer mortality rates had spread geographically outward beyond Laibin, Shuanglong, and Longchang, where coal mines were most concentrated.

## Discussion

This study provided updated lung cancer mortality rates in Xuan Wei during the last 5 years based on local death registration system (DRS) data. Using GIS-based spatial statistics, we mapped the spatial distributions of lung cancer mortality and visualized the population-level health risks of coal mines across Xuan Wei. In addition, the local population health risks were classified using a space-time GIS approach by overlaying pollutant concentration and population spatial distribution layers.

The basis of the different characteristics of Xuan Wei lung cancer mortality rates most likely resides in differences in environmental pollution exposure and the prevalence of risk factors. Therefore, town-level local maps of lung cancer mortality rates in these 5 years and intensity maps of the mortality and mine locations provided comprehensive clues.

The hotspot experienced a slight shrinkage from 2013 to 2015, but expanded and was surrounded by secondary hotspots of lower lung cancer mortality in 2015. The population-level health risks for those who live in villages surrounded by smoky coal mines showed stable geographical patterns, with the highest health risks in Shuanglong and Wanshui (type I) and the second highest health risks in Laibin (type II). The only spatiotemporal variation was the increased health risks in Luoshui in 2015 with the inward migration to this area. Geyi town witnessed a significant geographical concentration pattern and increased lung cancer mortality, although it was defined as a lower health risk (type IV) area over the study period.

The overall spatial correlations between coal mine distribution and lung cancer mortality were not significant but there were differences at the smaller spatial scale of town level compared to the overall spatial scale in Xuan Wei. The temporal correlativeness of lung cancer mortality in Xuan Wei was relatively steady between 2011 and 2015. The spatial correlation between coal mine distribution and lung cancer mortality in different areas of Xuan Wei was examined using the GWR model. The evidence showed that there was a significant spatial connection between coal mines and lung cancer mortality in geographically concentrated areas of the highest health risks (type I), while secondary significance was found at areas with medium (type III) and second-lowest health risks (type IV).

Furthermore, during the study period, the crude mortality rate and age-standardized rates of lung cancer in Xuan Wei dramatically increased in 2012 to 2014 compared to those in 2011. Compared to Xuan Wei overall, residents in Laibin have an increased risk of lung cancer, especially those over 35 years of age. Considering the average age of lung cancer in China is around 70+ years, efficient allocation of health interventions for at-risk population requires more accurate and complete information on the spatial associations of the intensity of the risk of lung cancer mortality in Xuan Wei. The age distribution indicated that the predominance of middle-aged cases in this area and part of this pattern might be due to the strong carcinogenetic elements of local smoky coal usage and the early age at which girls cook for their families. Girls generally begin to cook at around 9 years of age, and people from areas in Xuan Wei with higher lung cancer mortality rate frequently use smoky coal, while smokeless coal was more popular in areas in Xuan Wei that exhibited lower lung cancer rates [27]. Compared to the cancer crude and age-standardized rates in 2004–2005 [13], the mortality rates of lung cancer in the most recent period were similar for both men and women. The mortality of women Xuan Wei was almost eight times the average nationwide level during the same period and the sex ratio of mortality of 1.09 [28]. As the local people depend on solid fuels such as coal for cooking and heating in their daily lives, this links the further research on the carcinogenetic chemicals in the indoor coal smoke pollution.

In addition, the results of the present study identified Laibin town as a core area for future lung cancer research and intervention in Xuan Wei. Central “pocket-like areas” (PLAs) might be essential accumulation sites for coal combustion, with increased risks of air pollution [18]. In our town-level mortality comparison, the location of Laibin town matches that of a PLA. The ASR of lung cancer mortality in Laibin had increased from twice that of Xuan Wei during 2011–2012 to nearly four times that in 2013–2015 for men. It also increased one-fold among women during these two periods. Furthermore, during 2013–2015, the age-standard mortality rate for lung cancer mortality among those over 35 years of age increased faster than that for Xuan Wei. Patterns of death due to lung cancer in some regions with higher mortality risks still exist and the adverse effects act continually and accumulatively. This might be due to several large mines with abundant smoky coal around this town. Laibin also had the highest age-standard lung cancer mortality rate during 1990–2005 [29]. This indicated that the characteristics of regional concentration and carcinogenesis risk have not changed. It is obvious that the lung cancer problem in Xuan Wei has not been controlled, and it seems to be more serious in nearly all towns. Previous high-risk areas towns of Laibin and Rongcheng (Shuanglong, Wanshui, and a part of Hongqiao) still remain the top risk areas and some previously low health-risk are approaching high health-risk levels, especially Longchang, Haidai, and Baoshan (Fig. 11). There are several possible explanations for this observation, including (1) accumulation of carcinogens from coal combustion because of the geographical and spatial properties [29] and (2) increased communication and coal trading among nearby towns as part of the development of agricultural areas in China.

This research makes valuable contributions to related spatial epidemiology by adding the space-time correlation perspective of contextual effects and medical geography by exploring the fundamental Modifiable Areal Unit Problem (MAUP) at a smaller areal unit of villages regarding area-attribute analysis in geography, which has attracted increasing attention from public health realms. We utilized the towns as areal units as the town level has its advantages in conducting its environmental health-related policies and practices. In addition, although other elements such as socioeconomic status, healthcare accessibility might also be associated with lung cancer mortality, these factors were relatively evenly distributed among the towns in Xuan Wei.

However, this study also had several limitations. Our analysis of lung cancer mortality data was solely based on the Xuan Wei CDC death registration system (DRS) and there may have been underreporting or missing data for some mortalities. The total under-reported rate of deaths in Xuan Wei was 33.35% and there were no significant differences in separate sex or age groups [29]. Another potential limitation was the MAUP, in which the effects of area-based attributes on outcomes can be affected by the zoning scheme or geographic scale of the areal units [30]. Furthermore, topographical information and meteorological dynamics were not included in this study. Although topographical conditions are key influential factors of diseases, they generally contribute more to diseases associated with underground water and less to lung cancer [31]. Based on weather data collected from the governmental online database [32], 74.2% (1355 of 1826 days) of days in the 5-year study period had no wind and the number of days with winds from different directions did not vary significantly (37 days at maximum). The meteorological dynamics were more closely linked to human dynamics (e.g., human daily movement) and acute diseases in the short term [33]. Despite these limitations, the results of the present study are of value in exploring the static correlation between coal mine distribution and lung cancer mortality in small areal units over space and time. Our study provides data on the current lung cancer mortality demographic patterns, the spatial geographic accumulation risks, and areas for further carcinogen exploration and effective interventions within Xuan Wei, especially for communities with a high risk of lung cancer mortality.

## Conclusion

This study evaluated the most current information on lung cancer mortality in Xuan Wei, Yunnan, China and analysed the spatial-temporal health risks for lung cancer. The environmental risks and health results from coal mines demonstrated a geographically concentrated pattern around the geometric centre of Laibin, with some spatiotemporal variations. The spatiotemporal characteristics of cancer mortality in Xuan Wei lung are correlated with the accumulation of coal mines and their concentration around towns, especially large numbers of smoky coal mines. Subsequent intervention strategies for particularly toxic coal types require stronger proof of causality in further studies on the chemical characteristics of smoky coal. Specific exploration of the local environmental health risks related to indoor air pollution associated with the combustion of coal is also warranted. Moreover, the results of this study demonstrate the urgency of early screening for lung cancer among local people using smoky coal in high-risk areas.

## Abbreviations

DALYs: Disability-adjusted life-years
DRS: Death registration system
GIS: Geographic Information System
GWR: Geographically weighted regression
IARC: International Agency for Research on Cancer
ICD: International Classification of Disease
NCCR: National Central Cancer Registry
TASR: Truncated age-standardized rate
WHO: World Health Organization
WLS: Weighted least squares
WPRO: WHO Western Pacific Region
XWCDC: Xuan Wei Center for Disease Control and Prevention
